# Isolation and molecular characterization of prevalent Fowl adenovirus strains in southwestern China during 2015–2016 for the development of a control strategy

**DOI:** 10.1038/emi.2017.91

**Published:** 2017-11-29

**Authors:** Jing Xia, Ke-Chang Yao, Yue-Yue Liu, Guo-Jin You, Su-Yun Li, Ping Liu, Qin Zhao, Yi-Ping Wen Rui Wu, Xiao-Bo Huang, San-Jie Cao, Xin-Feng Han, Yong Huang

**Affiliations:** 1 College of Veterinary Medicine, Sichuan Agricultural University, Chengdu, Sichuan 611130, China

**Keywords:** epidemiology, FAdV serotype 4, FAdV serotype 8a, Fowl adenovirus, pathogenic, vaccine

## Abstract

Fowl adenovirus (FAdV) has caused significant losses in chicken flocks throughout China in recent years. However, the current understanding of the genetic and pathogenic characteristics of the FAdV epidemic in southwestern China remains poorly understood. In this study, a total of 22 strains were isolated from liver samples of diseased chickens from farms in southwestern China. Phylogenetic analysis based on the *hexon* loop-1 gene showed that the 22 isolates were clustered into four distinct serotypes: FAdV serotype 4 (FAdV-4) (86.4%, 19/22), FAdV-2 (4.5%, 1/22), FAdV-8a (4.5%, 1/22), and FAdV-8b (4.5%, 1/22). FAdV-4 was the predominant serotype in southwestern China. Pathogenicity testing showed that the FAdV-4 serotype strain CH/GZXF/1602 and FAdV-8a strain CH/CQBS/1504 were pathogenic to chickens, with mortality rates reaching as high as 80% and 20%, respectively. The primary clinical feature observed following infection with strain CH/GZXF/1602 (FAdV-4) was hepatitis-hydropericardium syndrome, and that of strain CH/CQBS/1504 (FAdV-8a) was inclusion body hepatitis. Conversely, the FAdV-2 serotype strain CH/GZXF/1511 and FAdV-8b serotype strain CH/CQBS/1512 was not observed to be pathogenic in chickens. Then, CH/GZXF/1602 (FAdV-4) was selected for the preparation of an inactivated oil-emulsion vaccine. Immune studies on Partridge Shank broilers showed that a single dose immunization at 17 days of age could not only protect against homologous challenge with virulent FAdV-4 but also provided protection against clinical disease following challenge with the heterologous FAdV-8b virulent strain until 70 days of age. The characterization of newly prevalent FAdV strains provides a valuable reference for the development of an efficacious control strategy.

## INTRODUCTION

Fowl adenoviruses (FAdVs) are nonenveloped viruses, with a linear, dsDNA genome belonging to the genus *Aviadenovirus.* The viruses could be clustered into five species (A–E), based on molecular structures,^1^ and 12 serotypes as determined via cross-neutralization tests.^2^ *Hexon* is the major protein of the adenovirus, and is known to contain the neutralizing epitope, which is serotype specific.^3, 4^ The serotype of FAdV is directly related to the *hexon* gene sequence.^5, 6, 7^ The most notable diseases associated with FAdV infection in chickens are the inclusion body hepatitis (IBH), hepatitis-hydropericardium syndrome (HHS) and gizzard erosions (GE). All 12 serotypes of FAdV are known to cause IBH.^8, 9^ IBH is typically observed in 3–5-week-old chickens, and is characterized by mortality approaching 10%. The primary lesions of IBH are a congested and enlarged liver with necrosis, petechial hemorrhage, and basophilic intranuclear inclusion bodies.^10, 11^ In contrast, HHS is primarily caused by FAdV serotype 4 (FAdV-4) strains.^12, 13, 14^ The mortality rate associated with HHS ranges from 30% to 70%, and the main lesions observed are IBH, nephritis and hydropericardium syndrome.^12, 15, 16^ With respect to GE, the major causative agents are FAdV-1 strains,^17, 18, 19^ and only a few cases have FAdV-4, FAdV-8 (FAdV-8a and -8b) and FAdV-11 strains been implicated. However, these cases are limited to experimental infections.^20, 21, 22^

HHS was first reported in the Angara Goth of Pakistan in 1987, and subsequent outbreaks have been recorded in many other countries, causing significant losses to the respective poultry industry.^12, 15, 23, 24^ HHS was not only observed in broilers, but also in pigeons and quail, although this happened only rarely. Until 2015, occurrences of IBH and/or HHS were hardly reported in southwestern China.^25^ However, since 2015, cases of suspected FAdV infection emerged and developed into epidemics in southwestern China, causing great economic losses in broiler and sometimes in hen production.^5, 6, 26^ Most of the cases that have been associated with FAdV showed severe HHS. The aim of this paper was to isolate the FadVs epidemic in southwestern China and to determine its molecular and pathogenic characteristics, thus preparing an efficacious oil-adjuvant inactivated vaccine. This study may provide novel insights into the epidemic and pathogenic character of FAdVs, and provide a novel control strategy for FAdV infection.

## MATERIALS AND METHODS

### Eggs and chickens

Specific pathogen-free chickens and embryos were obtained from the Beijing Merial Vital Laboratory Animal Technology Co. Ltd (Beijing, China). Healthy commercial, unvaccinated Partridge Shank broilers were obtained from De-Kang Agricultural and Livestock Technology Co. Ltd (Chengdu, China).

### Virus isolation

From 2015 to 2016, liver samples were collected from broiler or layer chickens suspected of FAdV infection in southwestern China. Samples were homogenized in phosphate-buffered saline (pH 7.0–7.4) at a ratio of 1:5–10. After three freeze–thaw cycles, the homogenates were centrifuged at 8000*g* for 10 min. Supernatants were collected for nucleic acid purification, polymerase chain reaction (PCR) detection and virus isolation. Total DNA extraction was performed using a DNA Extraction Kit (Invitrogen, Carlsbad, CA, USA) according to the manufacturer’s instructions. The presence of FAdVs in the supernatant was verified by PCR amplication of the *hexon* loop-1 gene using the primers *Hexon*-F (5′-CAA RTT CAG RCA GAC GGT-3′, position 144–161 nt) and Hexon-R (5′-TAG TGA TGM CGS GAC ATC AT-3′, position 1041–1021 nt).^27^ A touch-down PCR were performed using the following thermocycling protocol: 95 °C for 2 min, followed by 12 cycles of 95 °C for 30 s, 60 °C (−0.2 °C/cycle) for 30 s, 72 °C for 1 min, then 18 cycles of 95 °C for 30 s, 54 °C for 30 s, 72 °C for 1 min and 72 °C for 5 min A high fidelity DNA polymerase (PrimeSTAR Max DNA Polymerase; Takara Biotechnology (Dalian) Co. Ltd, Dalian, China) was used for the DNA amplification. The existence of other pathogens, including chicken infectious anemia virus, infectious bursal disease virus, Marek’s disease virus, avian leukosis vius and reticuloendotheliosis virus were assessed by PCR using previously published methods.^28, 29, 30, 31, 32^ Bacteria, such as *Escherichia coli* and *Salmonella*, were also isolated on blood agar plates.

Viruses were isolated via *in vitro* infection of chicken embryo kidney cells (CEKs). Briefly, monolayers of primary CEKs was seeded into 6-well plates from 18- to 20-day old specific pathogen-free chicken embryos, and were maintained in Dulbecco’s modified Eagle’s medium (Gibco, Grand Island, NY, USA) supplemented with 10% fetal bovine serum (Zhejiang Tianhang Biotechnology Co. Ltd, Huzhou, China) and incubated at 37 °C with 5% CO_2_.^33, 34^ The supernatants of liver lysates were filtered through a 0.22 μm filter, and 0.2 mL of the filtrate was overlayed onto the CEKs. Virus adsorption occurred at 37 ^o^C for 1 h. The culture supernatants were then discarded, and fresh Dulbecco’s modified Eagle’s medium supplemented with 2% fetal bovine serum was added to the CEKs. Supernatants and cells were harvested after 60 h incubation, and three blind passages were performed. Determination of the median tissue culture infectious doses (TCID_50_) of the FAdVs in CEKs was conducted.

### Phylogenetic analysis of the FAdV *hexon* gene

The PCR products of the *hexon* loop-1 gene were sequenced by the Sanggong Biotech Co. Ltd (Shanghai, China). Nucleotide sequences of the *Hexon* genes were aligned using the Editseq program in the Lasergene package (DNASTAR Inc., Madison, WI, USA), and were compared to the sequences of other reference FAdVs using MegAlign. The reference isolates included strains from the five species (FAdV A–E) with 12 serotypes (FAdV-1–7, -8a and -8b, -9–11), as well as duck adenovirus 1. A phylogenetic tree was created using the neighbor-joining method in MEGA version 7.0.14. Bootstrap values were determined from 1000 replicates of the original data.

### Virus pathogenicity

To investigate the pathogenicity of FAdV-2, FAdV-4, FAdV-8a and FAdV-8b field strains in chickens, representative strains from the four serotypes, CH/GZXF/1511 (FAdV-2), CH/GZXF/1602 (FAdV-4), CH/CQBS/1504 (FAdV-8a) and CH/CQBS/1512 (FAdV-8b) were chosen as the challenge viruses. Morbidity, mortality, clinical signs, viral DNA detection from cloacal swabs and tissue damage were the main indices used to evaluate the clinicopathological characterization of FAdVs.

**Figure 1:**
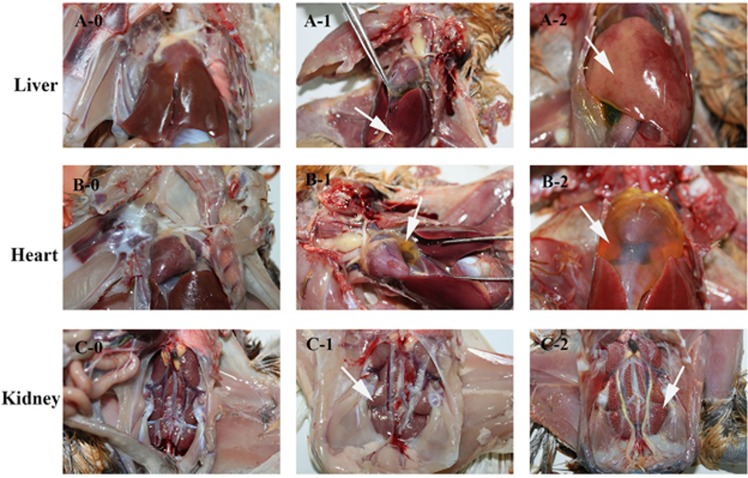
The score reference of gross lesions in liver, heart and kidney from chickens. The killed and dead birds of all groups were scored using this standard. Gross lesions of livers were scored as follow: 0 for normal (**A**-0), 1 for extensive focal lesions (**A**-1, white arrow), 2 for diffuse lesions (**A**-2, white arrow). Gross lesions of the heart were scored as follows: 0 for normal (**B**-0), 1 for v ≤2 mL of pericardial effusion (**B**-1, white arrow), and 2 for v >2 mL of pericardial effusion (**B**-2, white arrow). Gross lesions of kidneys were scored as follow: 0 for normal (**C**-0), 1 for slight swelling (**C**-1, white arrow) and 2 for severe swelling (**C**-2, white arrow).

Commercial 38-day-old unvaccinated Partridge Shank broilers (*n*=75) were randomly divided into five groups with 15 birds per group. In each group, five birds were used for histopathological analysis, and 10 birds were used for gross observations. Four groups of birds were subcutaneously injected with 10^4^ TCID_50_ of CH/GZXF/1511, CH/GZXF/1602, CH/CQBS/1504 or CH/CQBS/1512 strain, respectively, and the control group was inoculated with 0.2 mL phosphate-buffered saline. The chickens were housed in isolators under negative pressure. For histopathological diagnosis, the liver, heart and kidney of five birds including those succumbing to infection before 5 days postchallenge (d.p.c.), as well as some that were alive at 5 d.p.c. were sampled for analysis. The tissues were fixed in 10% neutral-buffered formalin. After 24 h of fixation, samples were processed, embedded in paraffin, stained with hematoxylin and eosin (HE) and observed using standard light microscopy. The morbidity and mortality were calculated for a period of 14 days, and dead birds were necropsied immediately. At 14 d.p.c., all animals were killed and necropsied to assess gross pathologic lesions. Gross lesions were observed in the liver, heart and kidney, and were scored as presented in Figure 1. Mean lesion scores were calculated for each group. In addition, cloacal swabs from both dead and surviving birds at 14 d.p.c in each group were collected and placed in 1 mL phosphate-buffered saline for viral DNA detection using the primers Hexon-F and Hexon-R as described above.

### Inactivated virus vaccine preparation

The FAdV-4 virulent strain, CH/GZXF/1602, was selected as the candidate vaccine strain and prepared as an oil-adjuvant inactivated virus vaccine. The TCID_50_ of virus was adjusted into 10^6^ TCID_50_/0.2 mL with phosphate-buffered saline. To inactivate FAdV, formaldehyde (0.1% in final product) was added to the cultures containing CH/GZXF/1602 strain at 37 ^o^C for 20 h, at which point the formalin was neutralized with sodium thiosulfate. Four percent of Tween-80 was then added into the inactivated virus as the aqueous phase, and the suspension was then emulsified into the oil phase (94% No. 7 white oil, 6% Span-80 and 2% aluminum stearate) at a ratio of 3:7 (v/v).

To assess the safety of the inactivated vaccine, ten 38-day-old Partridge Shank broilers were immunized subcutaneously with 0.3 mL oil-adjuvant inactivated virus vaccine. At 5 days postimmunization (d.p.i.), the liver, heart and kidney of five birds were sampled for histopathological scoring. At 14 d.p.i., all remaining animals were killed and necropsied for gross observation, as described previously.

### Immune efficacy of prepared and inactivated FAdV-4 vaccine

**Figure 2:**
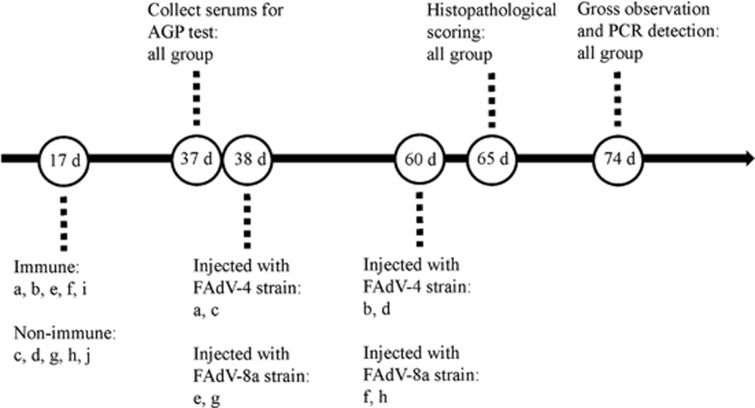
The immune and challenge schedule. Groups a and b: Birds were vaccinated at an age of 17 days and challenged with FAdV-4 strain at 38 days (a) and 60 days of age (b). Groups c and d: Birds were challenged with FAdV-4 strain at an age of 38 days (c) and 60 days (d). Groups e and f: Birds were vaccinated at an age of 17 days and challenged with FAdV-8a strain at 38 days (e) and 60 days (f). Groups g and h: Birds were challenged with FAdV-8a strain at an age of 38 days (g) and 60 days (h). Group i: Birds were vaccinated at an age of 17 days. Group j: Non-vaccination and non-challenge group.

To evaluate the protective efficacy of the inactivated FAdV-4 vaccine against homologous and heterologous FAdV challenge in unvaccinated Partridge Shank broilers, 17-day-old broilers (*n*=150) were randomly divided into 10 groups (named a–j). The immune and challenge schedule is shown in Figure 2.

Briefly, birds in Groups a, b, e, f and i were subcutaneously immunized with 0.3 mL oil-adjuvant inactivated virus vaccine at 17 days of age, birds in Group c, d, g and h were used as unvaccinated and challenge controls and birds in Group j were used as unvaccinated and unchallenged controls. Two time points postchallenge were selected to examine the protective efficacy of the vaccine in broilers. Serum samples of birds from all groups at 37 days of age were collected and tested for FAdV-specific antibodies by agar gel precipitation test.

Birds in Groups a and c were subcutaneously injected with 10^4^ TCID_50_ of homologous strain CH/GZXF/1602 (FAdV-4) at 38 days of age, while birds in b and d were injected at 60 days of age. Birds in group e and g were subcutaneously injected with 10^4^ TCID_50_ of heterologous strain CH/CQBS/1504 (FAdV-8a) at 38 days of age, whereas birds in Groups f and h were injected at 60 days of age. The morbidity and mortality were recorded during the study period. At 5 d.p.c., the liver, heart and kidney of five birds in each group were sampled for histopathological scoring. At 14 d.p.c., all remaining animals were killed and dissected for gross observation, and cloacal swabs were collected for PCR detection of virus as described above.

### Statistical analysis

The mean lesion scores were analyzed using an independent-samples *T*-test. A *P*-value of <0.05 was considered a statistically significant difference, *P*<0.01 was considered highly significant difference and *P*<0.001 was considered very highly significant difference.

### Ethics statement

All animal experiment such as generation of antiserum from specific pathogen-free chickens and immune protection tests of commercial vaccines were conducted complying with protocols approved by the Sichuan provincial Laboratory Animal Management Committee (Permit Number: XYXK (Sichuan) 2014-187) and the Ethics and Animal Welfare Committee of Sichuan Agricultural University. Humane end points were strictly adhered to over the entire experimental period. Birds that were either unable or unwilling to eat and/or drink during the study period were killed immediately, as well as all remaining birds at the end of the experiments were killed by cervical dislocation or by the administration of intravenous sodium pentobarbital (100 mg/kg) by a trained technician as approved by the Ethics and Animal Welfare Committee.

## RESULTS

### Virus isolation

A total of 53 clinical liver samples were collected from dead or diseased chickens displaying HHS or IBH from different chicken flocks located in southwestern China. This included the Si-chuan, Yun-nan, Gui-zhou and Chong-qing areas. A total of 28 samples were PCR positive for FAdVs, and a total of 22 unique FAdVs were isolated. Typical cytopathic effect of FAdVs on CEKs included cell rounding, aggregation, and an increase in the refractive index were observed during the passaging of FAdVs through CEKs. For the 28 samples, only two samples were found to be coinfected with chicken infectious anemia virus, and only 5 samples are positive for *E. coli* (data not shown). The case histories of local strains are listed in Table 1.

### Phylogenetic analysis of *hexon* gene

**Figure 3:**
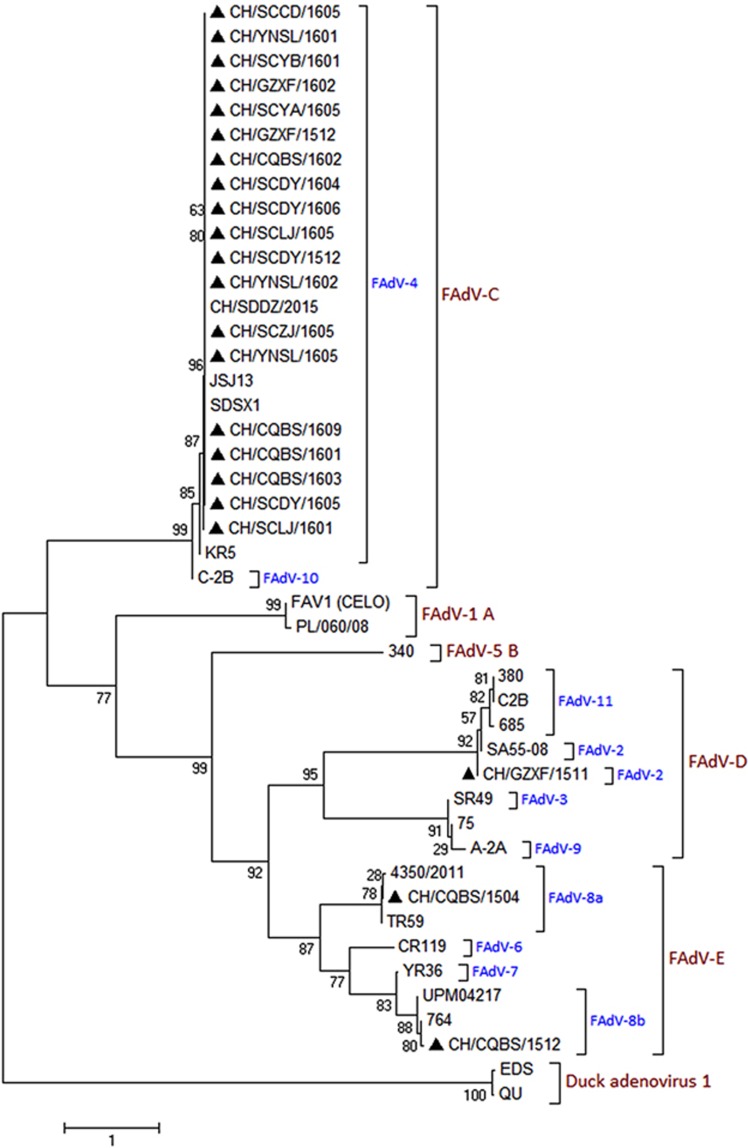
Phylogenetic analysis of the *hexon* loop-1 gene from 22 wild strains (filled triangles) and 22 reference strains of FAdVs, starting at the ^145^AAGT- and ending at -ACTA^1027^ (refer to FAdV-4 strains). The phylogenetic tree was constructed using MEGA version 7.0.14 with the neighbor-joining method and 1000 bootstrap replicates.

A portion of the *hexon* gene loop-1 sequences (896 bp) from the 22 FAdVs were determined and submitted to GenBank under the accession numbers MF055634–MF055655. Phylogenetic analysis based on the obtained sequences was used to classify these 22 FAdVs into four serotypes: FAdV-2, FAdV-4, FAdV-8a and FAdV-8b. A total of 19 isolates (CH/SCCD/1605, CH/YNSL/1601, CH/SCYB/1601, CH/GZXF/1602, CH/SCYA/1605, CH/GZXF/1512, CH/CQBS/1602, CH/SCDY/1604, CH/SCDY/1606, CH/SCLJ/1605, CH/SCDY/1512, CH/YNSL/1602, CH/SCZJ/1605, CH/YNSL/1605, CH/CQBS/1609, CH/CQBS/1601, CH/CQBS/1603, CH/SCDY/1605 and CH/SCLJ/1601) were included in the FAdV-4 serotype, sharing 97.4–100% nucleotide identity with *hexon* loop-1 gene sequences of other FAdV-4 reference strains. The strain CH/GZXF/1511 belonged to the FAdV-2 serotype and shared 99.8% nucleotide identity with the FAdV-2 reference strain. The strain CH/CQBS/1504 belonged to the FAdV-8a serotype, with 98.2–99.1% nucleotide identity with the FAdV-8a reference strains. Strain CH/CQBS/1512 clustered with the FAdV-8b serotype, sharing 98.4–99.3% nucleotide identity with the FAdV-8b reference strains. The phylogenetic tree constructed is presented in Figure 3.

### Virus pathogenicity

**Figure 4:**
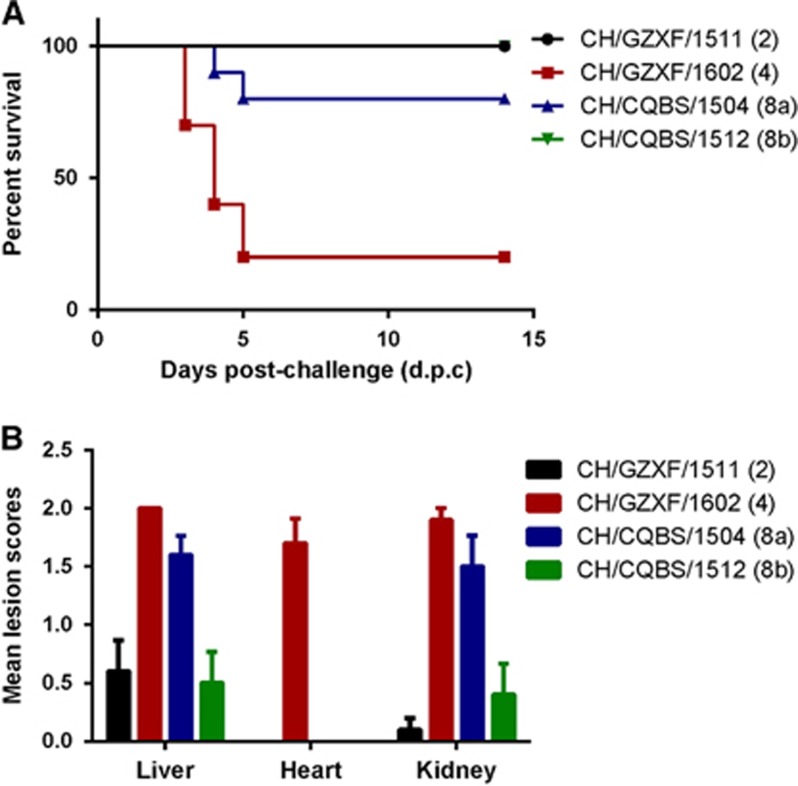
Pathogenicity of serotype-2, -4, -8a and -8b strains in Partridge Shank broilers. (**A**) Percent survival of chickens challenged by the four serotypes, respectively. The comments in brackets indicate the serotype of the challenge strains. (**B**) Mean lesion scores of chickens challenged by each of the four serotypes, respectively. Mean lesion scores reflected the mean scores of gross lesions.

Birds infected by both CH/GZXF/1602 (FAdV-4) and CH/CQBS/1504 (FAdV-8a) strains exhibited severe signs of mental depression and slight greenish discoloration associated with an increased urate component in the droppings from 2 to 8 d.p.c. For the group infected with CH/GZXF/1602 (FAdV-4), eight birds died between 3 to 5 d.p.c., with only two birds surviving the entire study period. The observed mortality rate was 80%. For the group infected with CH/CQBS/1504 (FAdV-8a), two birds died between 4-5 d.p.c., with eight birds surviving, giving a mortality rate of 20% (Figure 4A). The peak period of deaths in these two groups were on 4–5 d.p.c. For the other two groups infected with either FAdV-2 or FAdV-8b, no deaths occurred during the study, only mild mental depression was observed, and all animals exhibiting clinical signs recovered rapidly without intervention. No clinical signs and death were observed in the control group.

For gross pathologic lesions, the four groups exhibited marked differences with each other. Birds infected with CH/GZXF/1602 (FAdV-4) strain exhibited severe HHS and kidney enlargement. Mean lesion scores of liver, heart and kidney in this group were 2.0, 1.7 and 1.9, respectively. Intestinal hemorrhage was also observed in most of the infected birds. Birds infected with CH/CQBS/1504 (FAdV-8a) had severe IBH, kidney enlargement and intestinal hemorrhages, although there were no signs of hydropericardium syndrome observed. Mean lesion scores of liver, heart and kidney were 1.6, 0 and 1.5, respectively. However, for the other two groups infected by CH/GZXF/1511 (FAdV-2) and CH/CQBS/1512 (FAdV-8b), minor IBH and intestinal hemorrhages were observed, and none of the birds in these groups presented with hydropericardium syndrome. No lesions were observed in any of the control chickens. Mean lesion scores of each group are presented in Figure 4B.

For the histological lesions, the liver, heart and kidney of five birds from each group at 5 d.p.c were stained with HE and observed by light microscopy. In the CH/GZXF/1602 (FAdV-4)-infected group, severe diffuse lesions were observed in the liver, heart and kidney of birds. Lesions in the liver were characterized as diffuse fatty degeneration, congestion, intranuclear inclusion bodies and hemosiderin (Supplementary Figure S1). Lesions in the heart displayed epicardial incrassation, edema, congestion, hemorrhage, serous effusion and inflammatory cell infiltration (Supplementary Figure S2). Lesions in the kidney consisted of diffuse renal tubular epithelial cells, necrosis, falling, glomerular capsule dilation, congestion and hemosiderin (Supplementary Figure S3). In the CH/CQBS/1504 (FAdV-8a)-infected group, severe diffuse lesions were observed in the liver, including degeneration, congestion and intranuclear inclusion bodies s in hepatocytes (Supplementary Figure S4). In addition, kidney lesions consisted of diffuse renal tubular epithelial cells, coagulation, necrosis and falling, glomerular capsule dilation and congestion (Supplementary Figure S5). In contrast, there were no obvious pathological changes observed in the heart. In the CH/CQBS/1512 (FAdV-8b)-infected group, only minimal infiltration of inflammatory cells was observed in the liver portal area. Minor necrosis was observed in renal tubular epithelial cells in the kidney. There were no obvious pathologic changes observed in the heart. In the CH/GZXF/1511 (FAdV-2) and control groups, there were no FAdV-related histopathological changes in any of the tissues examined at 5 d.p.c.

For cloacal shedding of FAdVs, all of the birds (15/15) in CH/GZXF/1602 (FAdV-4) and CH/CQBS/1504 (FAdV-8a) challenge groups were positive. The FAdV-2 and FAdV-8b challenge groups had 40% (6/15) and 60% (9/15) of the animals shedding virus, respectively. No FAdV shedding was detected in the control group.

### Immune efficacy of prepared and inactivated FAdV-4 vaccine

The prepared inactivated vaccine was safe to birds, no clinical signs, mortality or FAdV-related pathologic changes were observed in specific pathogen-free chickens immunized with the inactivated vaccine.

All birds from five vaccinated groups (Groups a, b, e, f and i) seroconverted, whereas the birds from the five unvaccinated groups (Group c, d, g, h, and j) remained seronegative at 20 d.p.i in agar gel precipitation test.

**Figure 5:**
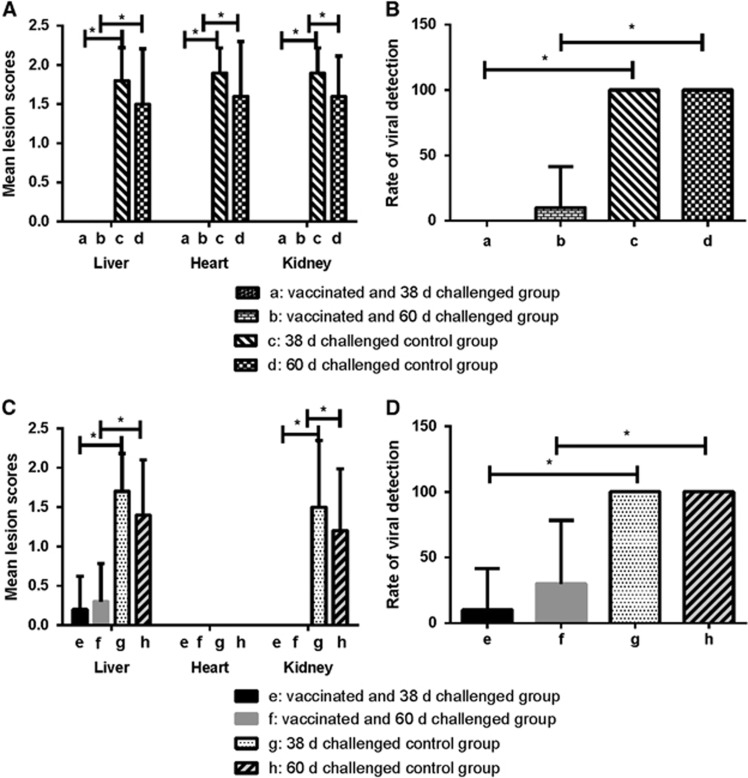
Protective efficacy of inactivated FAdV-4 vaccine against the FAdV-4 and FAdV-8a virulent challenge. (**A**) The mean lesions scores of vaccinated group (a and b) and unvaccinated group (c and d) challenged with the FAdV-4 strain. There were no lesions in the liver, heart and kidney in groups a and b, whereas birds in groups c and d showed severe lesions. (**B**) The viral detection rate in cloacal swabs of the vaccinated group (a and b) and unvaccinated group (c and d) challenged with the FAdV-4 strain. The viral detection rate in cloacal swabs of groups a and b were highly significantly different compared with groups c and d. (**C**) The mean lesion scores of the vaccinated groups (e and f) and unvaccinated groups (g and h) challenged with FAdV-8a strain. The vaccine significantly reduced the lesions caused by FAdV-8a. (**D**) The viral detection rate in cloacal swabs of vaccinated (e and f) and unvaccinated groups (g and h) challenged with FAdV-8a strain. The vaccine significantly reduced the rate of viral detection in cloacal swabs challenged with the FAdV-8a strain. *P<0.001 was considered very highly significant.

For birds in Groups a (challenged at 38 d) and b (challenged at 60 d), clinical signs, mortality, HHS and renal enlargement were not observed. For birds in Groups c and d, high mortality was observed (80% and 70%), as well as severe HHS. Independent-samples *T*-test showed that the difference of the mean lesion scores of liver, heart and kidney between a, b and c, d were very highly significant (*P*<0.001) (Figure 5A). Histopathology scoring showed that there were no apparent changes in liver, heart and kidney in birds from Group a. For birds in Group b, no changes were observed in heart and kidney, while one bird exhibited a small amount of inflammatory cell infiltration in the liver (Supplementary Figure S6). For Groups a, b, c, and d, 0%, 10%, 100%, and 100% cloacal swabs were detected as positive via PCR analysis, respectively. Statistical analysis revealed that the difference of cloacal shedding of FAdVs between groups a, b and c, d were very highly significant (*P*<0.001) (Figure 5B).

For birds in Groups e (challenged at 38 d) and f (challenged at 60 d), hydropericardium syndrome and renal enlargement were not observed, although slight IBH was observed in two birds from Group e and three from Group f. The mean lesion scores were 0.2 and 0.3, respectively (Figure 5C). Birds in Groups g and h presented with severe IBH and a 10–20% mortality rate was observed. Statistical analysis showed that the difference of the mean lesion scores of liver, heart and kidney between e, f and g, h were all very highly significant (*P*<0.001) (Figure 5C). Histological observation revealed a substantial amount of inflammatory cell infiltration, and a few intranuclear inclusion bodies in the liver of one bird from each of Groups e and f (Supplementary Figure S7). No histological changes were observed in the heart and kidney of birds in Groups e and f. In Groups e, f, g and h, 10, 30, 100 and 100% of cloacal swabs were detected as positive via PCR analysis, respectively. Statistical analysis revealed that the difference in cloacal shedding of FAdVs between Groups e, f and g, h were very highly significant (*P*<0.001) (Figure 5D).

In the control Groups i and j, there were no FAdV-related histopathological changes observed in the liver, heart and kidney, and FAdV was undetectable in the cloacal swabs at 14 d.p.c. (data not shown).

## DISCUSSION

In recent years, outbreaks of FAdVs have been frequently reported worldwide. Over the past 5 years, epidemics with mixed serotypes have been observed in different regions, such as FAdV-2, -11, -7 and -8 in Europe and North America,^7, 35, 36, 37^ FAdV-4 in Asia^5, 38, 39, 40, 41^ and FAdV-2 and -8b in South Africa.^42, 43^ The most notable diseases associated with FAdV infection in chickens are IBH, HHS and GE. There were no reports of severe HHS prior 2014 in China. However, recent outbreaks of HHS have been reported with high mortality rates in both broilers and duck.^38, 39^ Phylogenetic analysis of the *hexon* gene has revealed that strains circulating in China before 2014 and after 2015 had different ancestors, and that new strains circulating in China were derived from earlier Indian strains.^39^ The genome sequences of the contemporary strains have also been analyzed and clustered. This new pandemic FAdV-4 strain virus can be characterized by the notable nucleotide deletions in ORFs 19 and 27.^26, 38^ Sequence analysis of the *hexon* loop L1 gene has been routinely used to identify the serotype of field isolates. Therefore, we constructed a phylogenetic tree based on the *hexon* loop L1 sequences, and the results indicated that those 22 FAdVs were grouped primarily into four serotypes, with FAdV-4 strains (19/22) being the predominant viruses, further confirming that the FAdV-4 serotype was the prevalent type in China in the most recent 3 years.

Although all 12 serotypes of FAdVs have been associated with IBH, the majority of IBH cases in Japan, Canada and Australia were associated with the FAdV-8b serotype virus.^20, 21, 44, 45, 46^ Here, it was observed that the FAdV-8b strain, CH/CQBS/1512, caused only a mild clinical disease, with rapid recovery in Partridge Shank broilers. Unlike the CH/CQBS/1512 (FAdV-8b) strain, the FAdV-8a strain CH/CQBS/1504 showed severe IBH in broilers, characterized by focal hepatocellular necrosis, diffuse nephritis and mortality rates approaching 20%. To our knowledge, this is the first report in which the majority of IBH cases in China were associated with the FAdV-8a serotype. With respect to the pathogenicity of the CH/GZXF/1602 (FAdV-4) strain, our results indicated that the strain was more pathogenic than other strains from serotype 4, and the observed mortality was as high as 80% in broilers. All birds that succumbed to infection showed serious HHS. These results are consistent with previous studies which found that some FAdV-4 strains were highly pathogenic.^47^ The differences in pathogenicity of the different strains within the same serotype may be related to genetic differences between the strains or differential susceptibility of the chickens.^26, 38^

Various attempts have been made to control outbreaks of FAdV. Unfortunately, commercial vaccines with official approval are currently not available in the Chinese market and only several candidate vaccines with clinical trial approval have been used in clinical trials. However, there are a few vaccines against HHS and IBH on the market in other countries. Up to now, three types of vaccines, inactivated whole-cell vaccine, oral live attenuated vaccine and recombinant vaccine against HHS and IBH, have been reported.^25^ Preparation of the inactivated cell culture vaccine is easier and faster than both the live attenuated and recombinant vaccines, which is important in light of an ongoing outbreak. Several scholars have already reported that HHS and IBH can be effectively controlled through the use of inactivated cell culture vaccines.^48, 49, 50^ The cell culture inactivated oil-emulsion FAdV-4 vaccine is a promising candidate, providing effective heterologous protection.^50^ In the present study, a cell culture-inactivated oil-adjuvant FAdV-4 candidate vaccine was prepared. Safety testing showed that the vaccine was safe in 38-day-old chickens. Efficacy studies using a virulent virus challenge showed that a single dose in Partridge Shank broilers could provide excellent protection and clinical protection against both homologous (CH/GZXF/1602 FAdV-4) and heterologous (CH/CQBS/1512 FAdV-8b) challenge, respectively. HHS was not observed in any of the FAdV-4 challenged immunized birds. However, in immunized birds that were challenged with FAdV-8a, only a few chickens exhibited mild IBH, and histopathological observations showed minor inflammatory reactions in the liver. Not only that, but this candidate vaccine also significantly reduced virus shedding in both of the immunized groups compared to the challenge-control groups. In this study, the antigen load in the vaccine was adjusted to ensure that one shot of vaccine could provide the Partridge Shank broilers with protection until at least 70 days of age when the chickens would be sent to market, and one time of immunization could also significantly reduce the labor burden of farmers who inject the vaccine. We have also tested the immune efficacy of two other candidate vaccines with clinical trial approval from two companies, and the result showed that two immunizations with a interval of 14 days were required to provide protection against the challenge with virulent CH/GZXF/1602 (FAdV-4) and CH/CQBS/1504 (FAdV-8a) until 70 days of age (data not shown). The inactivated oil-emulsion FAdV-4 vaccine prepared for this study could be effective in preventing both occurrence and transmission of FAdV-related HHS and IBH in chicken flocks.

In conclusion, the newly emergent and prevalent FAdV-4 and FAdV-8a are the etiologic agents of FAdV infection in flocks from southwestern China in the most recent 3 years. Cases of HHS were associated with FAdV-4 strains, and IBH has been associated with FAdV-8a strains. One FAdV-4 serotype isolate CH/GZXF/1602 was selected for preparation of inactivated oil-emulsion vaccine and the immune efficacy of this vaccine on Partridge Shank broilers was promising, as a single shot was able to provide good protection against the challenge of homologous virulent FAdV-4 and heterologous virulent FAdV-8b strains. The characterization of newly prevalent FAdV strains provides a valuable reference for the development of an efficacious control strategy.

## Figures and Tables

**Table 1:** Information for the 22 FAdV isolates in this study

**FAdV strains**	**Isolates date**	**Production type**	**Age (day)**	**Isolated location**	**Symptoms phenotype HHS** **and IBH**	**Accession number**	**Serotype**
CH/CQBS/1504	2015.04	Broiler	26	Chongqing	b	MF055634	FAdV-8a
CH/GZXF/1511	2015.11	Broiler	46	Guizhou	a	MF055635	FAdV-2
CH/GZXF/1512	2015.12	Broiler	51	Guizhou	b	MF055636	FAdV-4
CH/CQBS/1512	2015.12	Broiler	43	Chongqing	b	MF055637	FAdV-8b
CH/SCDY/1512	2015.12	Broiler	32	Sichuan	a	MF055638	FAdV-4
CH/SCLJ/1601	2016.01	Layer	118	Sichuan	a	MF055639	FAdV-4
CH/SCYB/1601	2016.01	Layer	135	Sichuan	a	MF055640	FAdV-4
CH/YNSL/1601	2016.01	Broiler	70	Yunnan	a	MF055641	FAdV-4
CH/CQBS/1601	2016.01	Broiler	21	Chongqing	a	MF055642	FAdV-4
CH/GZXF/1602	2016.02	Broiler	56	Guizhou	a	MF055643	FAdV-4
CH/YNSL/1602	2016.02	Broiler	56	Yunnan	a	MF055644	FAdV-4
CH/CQBS/1602	2016.02	Broiler	67	Chongqing	a	MF055645	FAdV-4
CH/CQBS/1603	2016.03	Broiler	52	Chongqing	a	MF055646	FAdV-4
CH/SCDY/1604	2016.04	Layer	126	Sichuan	a	MF055647	FAdV-4
CH/YNSL/1605	2016.05	Broiler	30	Yunnan	a	MF055648	FAdV-4
CH/SCCD/1605	2016.05	Broiler	35	Sichuan	a	MF055649	FAdV-4
CH/SCYA/1605	2016.05	Broiler	21	Sichuan	a	MF055650	FAdV-4
CH/SCDY/1605	2016.05	Broiler	67	Sichuan	a	MF055651	FAdV-4
CH/SCZJ/1605	2016.05	Broiler	35	Sichuan	a	MF055652	FAdV-4
CH/SCLJ/1605	2016.05	Broiler	28	Sichuan	a	MF055653	FAdV-4
CH/SCDY/1606	2016.06	Broiler	37	Sichuan	a	MF055654	FAdV-4
CH/CQBS/1609	2016.09	Broiler	28	Chongqing	a	MF055655	FAdV-4

Abbreviations: Fowl adenovirus, FAdV; hepatitis-hydropericardium syndrome, HHS; inclusion body hepatitis, IBH.
