# Supplementary material for: Isolation and molecular characterization of prevalent Fowl adenovirus strains in southwestern China during 2015–2016 for the development of a control strategy
Source: Emerg Microbes Infect. 2017 Nov 29;6(11):e103–. doi: 10.1038/emi.2017.91 (PMC5717092; doi:10.1038/emi.2017.91)
Supplement: Supplementary Figure 2 [file emi201791x2.pdf]

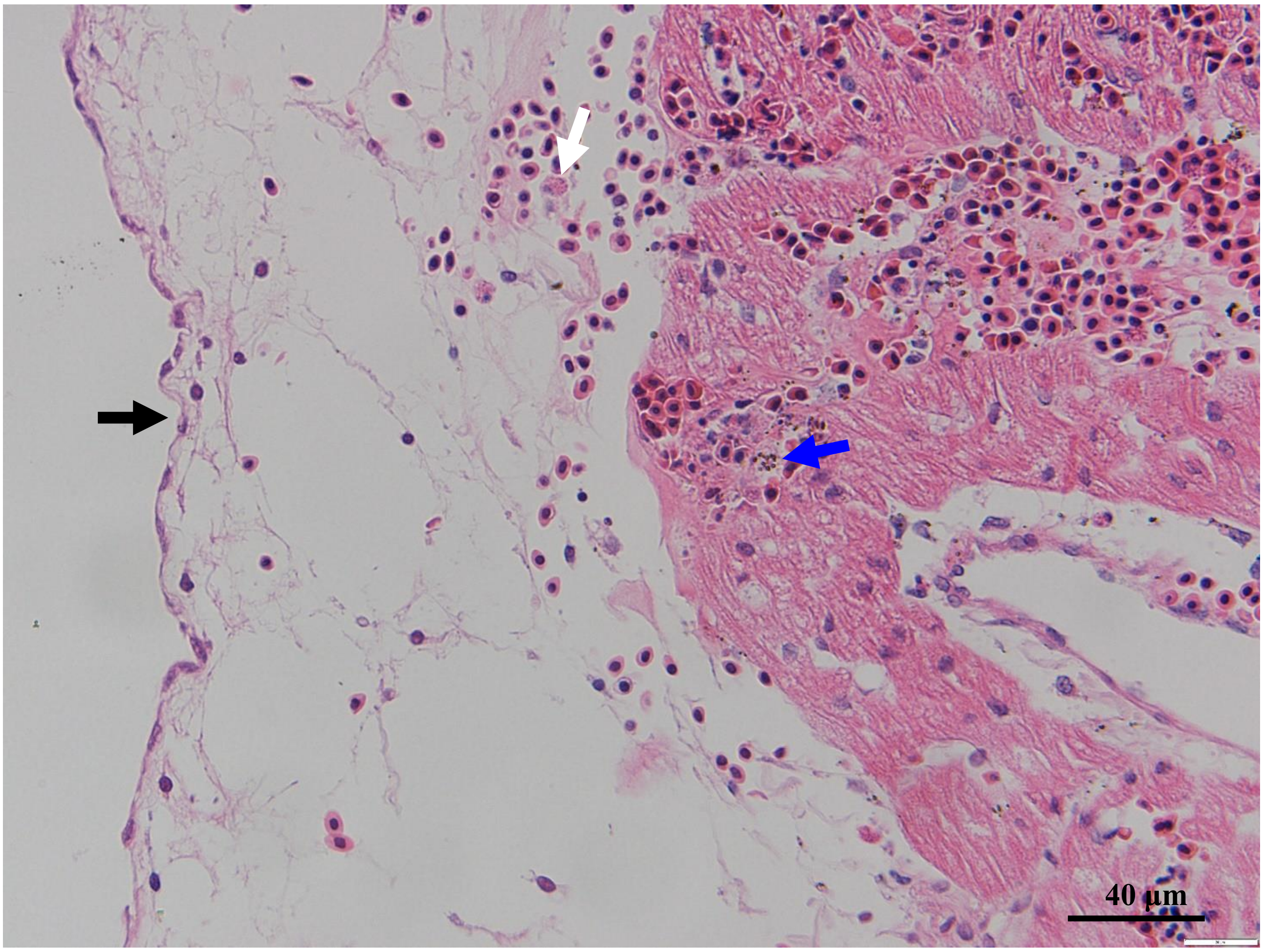

Supplementary Figure 2: Heart lesions in a chicken challenged with CH/GZXF/1602 (FAdV-4) at 5 d.p.c. Epicardium incrassation (indicated with a black arrow), edema, congestion, hemorrhage, inflammatory cell infiltration (indicated with a white arrow), serous effusion, and hemosiderin (indicated with a blue arrow).
