# Supplementary material for: Isolation and molecular characterization of prevalent Fowl adenovirus strains in southwestern China during 2015–2016 for the development of a control strategy
Source: Emerg Microbes Infect. 2017 Nov 29;6(11):e103–. doi: 10.1038/emi.2017.91 (PMC5717092; doi:10.1038/emi.2017.91)
Supplement: Supplementary Figure 5 [file emi201791x5.pdf]

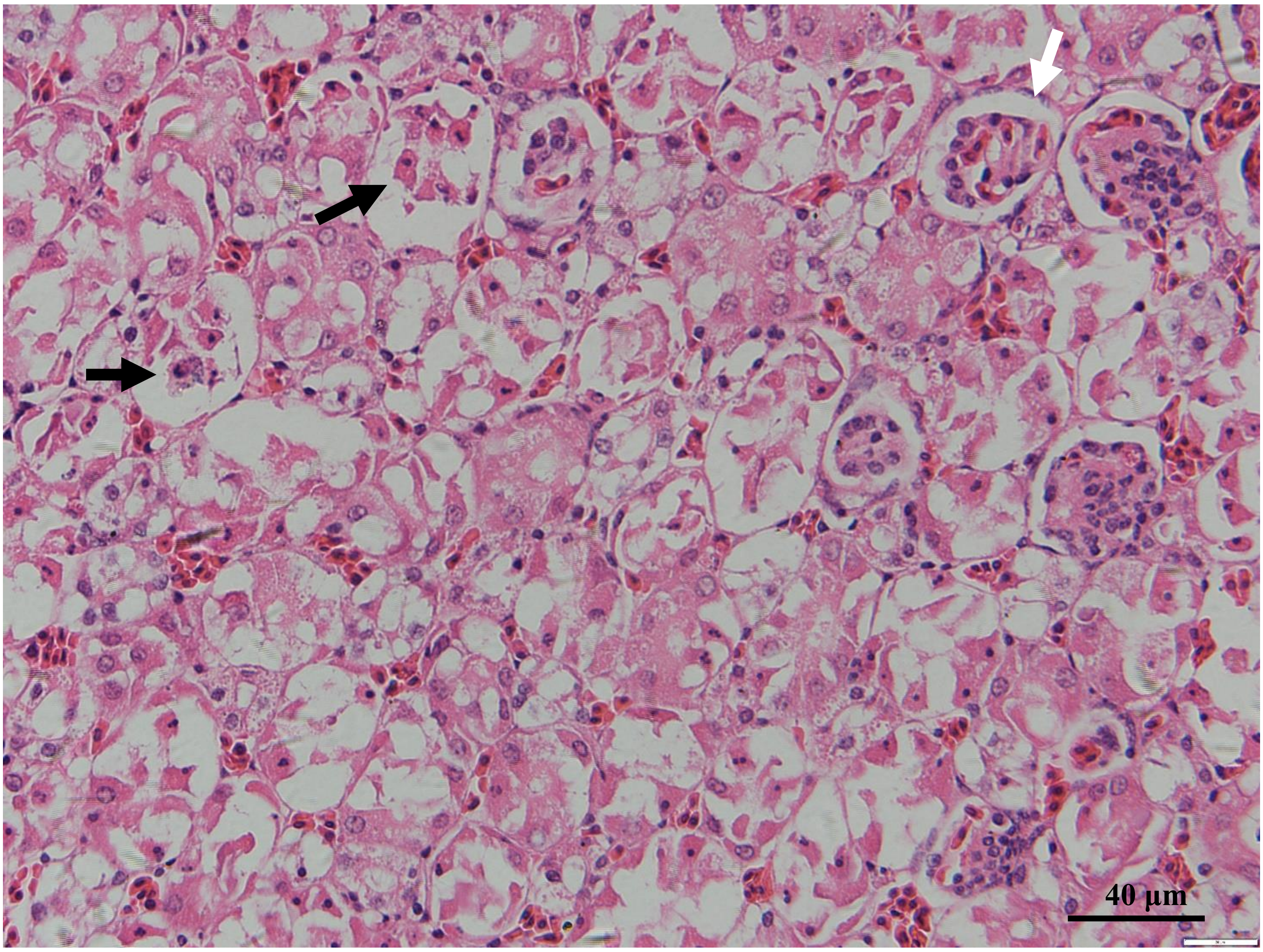

Supplementary Figure 5: Kidney lesions of a chicken challenged with CH/CQBS/1504 (FAdV-8a) at 5 d.p.c. Diffuse renal tubular epithelial cell coagulation necrosis and falling (indicated with black arrows) and glomerular capsule dilation (indicated with a white arrow).
